# Expression of Viral microRNAs in Serum and White Blood Cells of Cows Exposed to Bovine Leukemia Virus

**DOI:** 10.3389/fvets.2020.536390

**Published:** 2020-09-22

**Authors:** Eduardo Casas, Hao Ma, John D. Lippolis

**Affiliations:** National Animal Disease Center, Agricultural Research Service (ARS), United States Department of Agriculture (USDA), Ames, IA, United States

**Keywords:** BLV, cattle, microRNA, serum, white blood cells

## Abstract

Bovine leukemia virus (BLV) affects the health and productivity of cattle. The virus causes abnormal immune function and immunosuppression. MicroRNAs (miRNAs) are involved in gene expression, having been associated with stress and immune response, tumor growth, and viral infection. The objective of this study was to determine the expression of circulating miRNAs produced by BLV in animals exposed to the virus. Sera from 14 animals were collected to establish IgG reactivity to BLV by ELISA, where seven animals were seropositive and seven were seronegative for BLV exposure. White blood cells (WBC) from each animal were also collected and miRNAs were identified by sequencing from sera and WBC. The seropositive group had higher counts of BLV miRNAs when compared to seronegative group in sera and WBC. Blv-miR-1-3p, blv-miR-B2-5p, blv-miR-B4-3p, and blv-miR-B5-5p were statistically significant (*P* < 0.00001) in serum with an average of 7 log2 fold difference between seropositive and seronegative groups. Blv-miR-B1-3p, blv-miR-B1-5p, blv-miR-B3, blv-miR-B4-3p, blv-miR-B4-5p, blv-miR-B5-5p were statistically significant (*P* < 1.08e^−9^) in WBC with an average of 7 log2 fold difference between the seropositive and the seronegative groups. Blv-miR-B2-3p and blv-miR-B2-5p were also statistically significant in WBC (*P* < 2.79e-17), with an average of 27 log2 fold difference between the seropositive and the seronegative groups. There were 18 genes identified as being potential targets for blv-miR-B1-5p, and 3 genes for blv-miR-B4-5p. Gene ontology analysis indicated that the target genes are mainly involved in the response to stress and in the immune system process. Several of the identified genes have been associated with leukemia development in humans and cattle. Differential expression of genes targeted by BLV miRNAs should be evaluated to determine their effect in BLV replication.

## Introduction

Bovine Leukemia virus (BLV) belongs to the *Retroviridae* family, produces enzootic bovine leukosis, and is associated with subclinical infections in cattle (1–3). The disease has been characterized as having three stages: asymptomatic or aleukemic, persistent lymphocytosis, and leukemia or lymphoma (3, 4). Approximately 83% of the cows in dairy herds, and 39% of cattle at slaughter are infected with BLV (2, 3). No commercial vaccine is available and control of the virus is by culling positive animals (3). Infection with BLV has been associated with higher culling rates and shorter herd life, contributing to the cost of the production system. It has been estimated that the annual cost of subclinical BLV infection is more than $6,400, with a 50% prevalence in a 100 herd of cows in the mid-Atlantic region (1).

MicroRNAs (miRNAs) are small non-coding RNA molecules ranging in size from 21 to 25 nucleotides in length, which regulate gene expression by altering translation (5–7). Host miRNAs have been proposed as biomarkers for exposure to pathogens in humans (8, 9), and in livestock (10–12). Cattle exposed to different pathogens has shown differential expression of various miRNAs (13–15).

MiRNAs have been identified in what was thought to be an inactive region of the BLV genome (16, 17), and their activity in developing tumoral cells has also been identified (18–21). However, the expression of BLV miRNAs has not been established; therefore, the objective of this study was to determine the expression of circulating miRNAs produced by BLV in animals exposed to the virus.

## Materials and Methods

Animals and laboratory techniques have been previously described (22). Following is a brief description of the procedures used.

### Animals

Fourteen Holstein female cattle were used to collect samples at the National Animal Disease Center, in Ames, IA, United States. All animals included in the study were considered healthy according to the attending veterinarian. Samples came from 3 heifers and 11 cows with at least one calving and at mid-lactation. Jugular venipuncture was used to collect blood samples in PAXgene tubes (PreAnalytiX GmbH, Hombrechtikon, Zurich, Switzerland), from which white blood cells (WBCs) were obtained. Samples were kept at room temperature for 2 h and refrigerated (4°C) before centrifugation (3,000 g for 10 min) to collect serum and WBC. Management of animals was according to the protocol approved by the Institutional Animal Care and Use Committee of the National Animal Disease Center, in Ames, IA, United States.

### ELISA

An additional blood sample was collected via jugular venipuncture in serum separator vacutainer tubes (SST, TM BD, Franklin Lakes, NJ, United States). Tubes were incubated (37°C for 30 min) and centrifuged (1,250 g for 30 min). Sera were stored at −80°C until processed. Sera were used to determine IgG response to BLV, using the IDEXX Leukosis Serum X2 Ab kit (Idexx Laboratories, Westbrook, ME, United States). Samples were determined positive according to manufacturer's directions. A positive was established if the sample to positive ratio (S/P) was >115%. The sample to positive ratio was the ratio of the difference between the optical density 450 nm of the sample minus the optical density 450 nm of the negative control, divided by the difference between the optical density 450 nm of the mean positive control minus the optical density 450 nm of the negative control. Seven animals were seronegative (negative group), and seven were seropositive (positive group).

### Small Non-coding RNA Isolation

Total RNA was extracted from serum and WBC samples using the MagMAXTM mirVanaTM Total RNA Isolation Kit (Life Technologies, Carlsbad, CA, United States) and total RNA was eluted in 100 μL of RNase-free water. The concentration and quality of small RNAs was determined using an Agilent 2100 Bioanalyzer Small RNA chip (Agilent Technologies, Santa Clara, CA, United States).

### Library Preparation and Sequencing

Six microliters (6 μL) of small RNA from each sample was used to prepare each library using the NEBNext Multiplex Small RNA Library Prep Kit (New England BioLabs, Ipswich, MA, United States) and 14 indexed primers, which gave all samples a unique identifier. Concentration of libraries and purification was done using the QIAquick PCR purification kit (QIAGEN, Germantown, MD, United States). Each library was loaded on an Agilent 2100 Bioanalyzer High Sensitivity DNA chip (Agilent Technologies, Santa Clara, CA, United States) to establish quality and quantity of each library between 135 and 170 base pairs. Thirty nanogram of each library was pooled (14 libraries in the pool) and size selected using AMPure XP beads (Beckman Coulter, Indianapolis, IN, United States). After selecting for size, the pools were concentrated using the QIAquickPCR purification kit (QIAGEN, Germantown, MD, United States) and eluted in RNase-free water. To determine the concentration of each pool, an Agilent 2100 Bioanalyzer High Sensitivity DNA chip (Agilent Technologies, Santa Clara, CA, United States) was used. Each pool was stored at −20°C until sequencing. The pool was sequenced as single-end 50 base pair reads using the Illumina HiSeq 3000 System (Illumina, San Diego, CA, United States).

### Data Analysis

The BLV reference genome (GenBank assembly accession: GCA_000853665.1) was downloaded from NCBI (ViralProj14916). The BLV miRNA precursor and mature sequences were downloaded from miRBase (Release 22.1). The quality of raw sequencing reads were evaluated by FastQC (23), and were cleaned with cutadapt (24). The raw and normalized BLV miRNA reads were counted by using miRDeep2 (version 2.0.0.8) (25). The counted BLV miRNA raw reads were then used to identify differentially expressed miRNAs by running DESEQ2 package (26). Target genes of the differentially expressed miRNAs were separately predicted by miRanda (Total score ≥450 and max energy ≤-20 kcal/Mol) and PITA (Energetic score ≤-20 kcal/Mol) (27, 28). Gene ontology of the target genes were analyzed by using OmicsBox (www.biobam.com). Sequences are available on the NCBI SRA under BioProject accession number PRJNA378560.

## Results

A total of 144,343,706 reads were mapped to bovine miRNAs from serum and WBC. There were 313,261 sequences that mapped to viral miRNAs (Table 1). Of these, 32,538 mapped to miRNAs in serum and 280,723 to miRNAs in WBC. Sequences were identified for all known mature BLV miRNAs, with the exception of blv-miR-B3-5p. The remaining sequences were mapped to bovine miRNAs and excluded from further analysis.

**Table 1:** Number of sequences for each miRNA identified in serum and white blood cells (WBC), in the seronegative and seropositive groups.

**miRNA**	**Serum**		**WBC**		**Total**
	**Seronegative**	**Seropositive**	**Seronegative**	**Seropositive**	
blv-miR-B1-3p	1	0	714	116,119	116,834
blv-miR-B1-5p	0	0	2	510	512
blv-miR-B2-3p	16	4,989	0	26,711	31,716
blv-miR-B2-5p	22	6,532	0	41,030	47,584
blv-miR-B3-3p	0	440	63	9,520	10,023
blv-miR-B4-3p	30	6,963	260	43,875	51,128
blv-miR-B4-5p	1	71	2	285	359
blv-miR-B5-3p	0	0	2	31	33
blv-miR-B5-5p	47	13,426	208	41,391	55,072
Total	117	32,421	1,251	279,472	313,261

**Figure 1:**
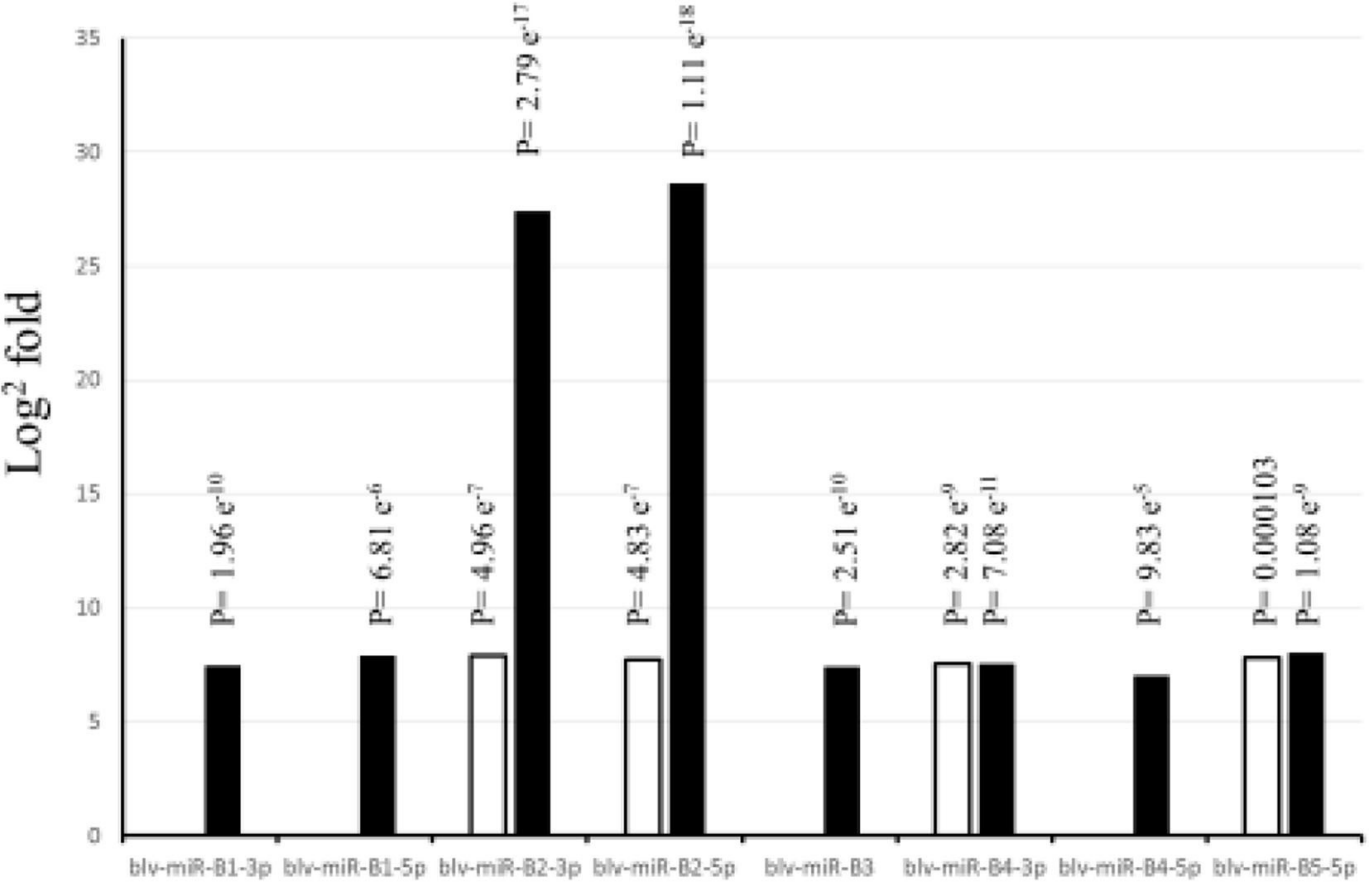
Differences in expression (Log^2^ fold) between cows positive and negative to exposure to bovine leukemia virus. White bars indicate expression in serum, and black bars indicate expression in white blood cells. *P*-values are adjusted and equivalent to false discovery rate (FDR).

Figure 1 shows the significant (*P* < 0.05) log2 fold differences between the positive and the negative groups. For all significant BLV miRNAs, the positive group had greater number of sequences than the negative group. Blv-miR-B5-3p was not significant in serum or in WBC. In serum, blv-miR-B2-3p, blv-miR-B2-5p, blv-miR-B4-3p, and blv-miR-B5-5p, were significant and had an expression difference between the positive and the negative groups ranging from 7 to 8 log2 fold. In WBC, blv-miR-B2-3p and blv-miR-B2-5p had a 27 and 29 log2 fold difference between the two groups, respectively. The other significant BLV miRNAs in WBC had a difference in expression ranging from 7 to 8 log2 fold, which is similar to those observed in serum.

The gene ontology analysis of the genes targeted by blv-miR-B1-5p and blv-miR-B4-5p, as involved in response to stress and immune system process pathways (Table 2). No other BLV miRNA was involved in these pathways. Both BLV miRNAs target different genes in both pathways. Blv-miR-B1-5p targets 15 different genes in the response to stress pathway and 9 genes in the immune system process pathway; however, *C4A, COLEC12, GPS2*, and *TLR9*, are involved in both pathways. Blv-miR-B1-5p targets a total of 20 genes in both pathways. Blv-miR-B4-5p targets 3 genes, *ZFYVE26* in the response to stress pathway, *FAM20C* and *MADCAM1* in the immune system process pathway.

**Table 2:** Putative genes targeted in the host by blv-miR-B1-5p and blv-miR-B4-5p, identified as being involved in the response to stress and immune system processes.

**miRNA**	**Gene ontology term**	**Genes**		
Blv-miR-B1-5p	Response to stress	*AJAP1*	***COLEC12***	*HYOU1*
		*AXIN1*	*EDAR*	*PAXIP1*
		*AXIN2*	*ERCC4*	*SH3RF3*
		***C4A***	*FGFR10P2*	*SLX4*
		*CCNK*	***GPS2***	***TLR9***
	Immune system process	***C4A***	***COLEC12***	*IRF2BP2*
		*CD3E*	*GAB3*	*PTK2B*
		*CD74*	***GPS2***	***TLR9***
Blv-miR-B4-5p	Response to stress	*ZFYVE26*		
	Immune system process	*FAM20C*	*MADCAM1*	

*In bold are genes targeted by both miRNAs*.

## Discussion

In a previous study, we identified differentially expressed transfer RNA fragments produced by the animal as a response to the exposure to BLV (22).

In the present study, the same animals were used to detect differentially expressed microRNAs produced by the BLV in cattle.

It has been established that BLV miRNAs modify gene expression of the host, promoting pathogenicity (19, 21). The comparison in expression of these miRNAs between positive and negative cattle groups is absent. Therefore, it was important to establish their expression to ascertain the potential role these miRNAs may play in establishing a BLV infection.

Blv-miR-B2-3p and blv-miR-B2-5p had the greatest differences in expression in WBC, compared to other BLV miRNAs. These miRNAs were not found to be involved in the response to stress or the immune system process pathways. Blv-miR-B2-5p was uncorrelated with other miRNAs in a study where BLV seroconversion status was compared (21). Blv-miR-B2-5p was found to be associated with the expression of *RPTN*. This gene is involved in cell differentiation and is down-regulated in patients with human papillomavirus (29). It is probable that blv-miR-B2-5p may down-regulate *RPTN* during a BLV infection. Despite being the BLV miRNAs with the greatest differences in expression between the positive and negative groups, they seem to be involved in processes other than the response of the animal to a BLV infection. Blv-miR-B2-3p has not been evaluated (21).

Blv-miR-B1-5p and blv-miR-B4-5p were associated with response to stress and immune systems processes, and were only significantly differentially expressed in WBC. It is possible that these BLV miRNAs are released in serum due to conclusion of their function within the cell. Additional studies would need to establish the reason for the release of BLV miRNAs in serum.

Genes found to be influenced by blv-miR-B1-5p have been observed to be related with leukemia in humans. The genes *C4A, GPS2*, and *COLEC12*, are involved in response to stress and in immune system processes. In addition, dysregulation of *C4A*, and *GPS2*, are also known to be involved in the development of leukemia in humans when they are dysregulated (30, 31). The gene *COLEC12* produces a receptor that mediates the cross-talk between dendritic cells and gastric stromal cells and has been associated with the development of gastric cancer patients when deregulated (32). BLV miRNAs seem to dysregulate the expression genes to proliferate in cattle.

Toll-like receptors are a family of conserved glycoproteins that play a role in the innate immune system. The function is to sense the presence of a pathogen and initiate the immune response (33). The Toll-like receptor-9 (*TLR9*) was identified as being a target for blv-miR-B1-5p. The *TLR9* gene is upregulated in cattle with a BLV high proviral load, compared to animals with a low proviral load, as well as an increase in messenger RNA for the protein produced by this gene (34, 35). It is unclear if the role of the blv-miR-B1-5p is to down-regulate the expression of this gene, given that in a BLV infection, *TLR9* is overexpressed in cattle (34, 35). Further studies would need to be pursued to understand the role of blv-miR-B1-5p in a BLV infection in cattle.

The Wnt signaling pathway is composed of intercellular signaling proteins that regulate proliferation, cell division, and other processes relevant to cellular survival (36). The *AXIN2* gene is the universal target of this pathway (37) and is dysregulated in leukemia patients (37, 38). It is probable that blv-miR-B1-5p dysregulates *AXIN2* to produce leukemia in cattle.

Additional genes identified as being target of blv-miR-B1-5p have also been associated with different types of leukemia in humans. Structural chromosome rearrangements, specifically chromosome translocations that create fusion genes encoding chimeric proteins, characterize different types of leukemia. Genes involved in these fusion genes were identified as targets for blv-miR-B1-5p. Patients with a subtype of acute myeloid leukemia (AML), known as acute promyelocytic leukemia, show the fusion of the retinoic acid receptor alpha (*RARA*), with the promyelocytic leukemia gene. The gene *IRF2BP2* is a target for blv-miR-B1-5p. In acute lymphoblastic leukemia (ALL) cases, the gene *PTK2B*, which is a target gene for blv-miR-B1-5p, has been identified as forming a fusion gene with *KDM6A, STAG2*, or *TMEM2* (39, 40). In the case of stem cell leukemia-like syndrome, a fusion gene between *FGFR1* and *FGFR1OP2* characterize this condition in humans (41–43). The gene *FGFR1OP2* was also identified as a target for blv-miR-B1-5p. The role of blv-miR-B1-5p is unclear in the fusion of the genes including *IRF2BP2, PTK2B*, and *FGFR1OP2*, if any. Further studies will need to establish if blv-miR-B1-5p plays a part in the development of fusion genes in leukemia cases in cattle.

The product of the gene *CD74* is known to be involved in the defense mechanism of AML cells. This gene is a signaling receptor for the major histocompatibility complex type II, involved in antigen presentation, and its expression has been linked to cell survival pathways. Its expression has been associated with poor survival in patients with AML (44, 45). Expression of this gene assists in the defense mechanism of leukemia cells in the case of AML. This gene was identified as a target for blv-miR-B1-5p. It is possible the miRNA may influence the expression of *CD74* in AML patients.

Overexpression of the *SLX4* gene has been observed in the B-cell chronic lymphocytic leukemia (BCLL). B-cell chronic lymphocytic leukemia is characterized by lengthening of the telomeres. *SLX4* is involved in the process known as alternative telomere lengthening, which characterizes BCLL (46). Similarly, *SLX4* has also been associated with onset of Fanconi anemia, which is also characterized by telomere lengthening (47). This gene is a target by blv-miR-B1-5p, and has the potential to be associated with the development of both diseases.

Limited literature is available indicating the association of *SH3RF3, PAXIP1*, and *CD3E*, targeted by blv-miR-B1-5p. As part of a panel of 6 genes, *SH3RF3* is used to establish the prognosis of patients affected with ALL. When *SH3RF3* is expressed, in conjunction with *CAMSAP1* and *PCGF6*, the outcome of ALL is favorable; however, when this genes is not expressed in ALL patients, the outcome of the disease is less than desirable (48). Blv-miR-B1-5p, may have a role in inhibiting expression of *SH3RF3* to establish leukemia in cattle. The hematopoietic stem cells (HSC) are responsible in providing the organism with mature blood cells. The HSC reside in bone marrow pockets in the interphase of the bone and bone marrow. The *PAXIP1* or *PTIP* genes are epigenetic regulators responsible for maintaining the bone marrow pockets. Downregulation of *PAXIP1* results in downregulation of HSC. Downregulation of *PAXIP1* has also been associated with downregulation of leukemia cells (49). The role of blv-miR-B1-5p in the regulation of *PAXIP1* is unclear given that downregulation of *PAXIP1* would result in a downregulation of leukemia cells. It has been established that *CD3E* is overexpressed in patients with T-cell ALL (50), and that patients with this condition and that a remission of the disease can be obtained with an anti-CD3E recombinant immunotoxin (51). Potentially, blv-miR-B1-5p has a role in the overexpression of *CD3E* in T-cell ALL patients; however, further studies would be needed to establish this effect. A similar process could be occurring for BLV infection in cattle.

Genes targeted by blv-miR-B4-5p have not been identified as playing a role in the development of leukemia in humans. Variants in the *ZFYVE26* gene have been associated with colorectal cancer (52), variants in the *MADCAM1* gene have been associated with multiple sclerosis (53), and variants in the *FAM20C* gene have been associated with development of Raine syndrome in humans (54). Although they are classified as being involved in the response to stress and immune system process pathways, there is no evidence suggesting their role in the development of leukemia. The role of these genes in processes associated with leukemia, and their relationship with BLV miRNAs, should be determined.

The sequence count for each BLV miRNA was greater in the positive group, compared to the negative group as expected; however, there were BLV miRNA sequences found in animals considered negative. This is possible given that animals were classified based on an ELISA. It is probable that animals with a negative ELISA, resulted with a low count of BLV miRNAs because they were exposed to the virus but had yet to develop an immune response high enough for a positive ELISA result. A longitudinal analysis could have established if animals considered seronegative at sampling seroconvert in future samplings.

There are differences in count number of sequences between serum and WBC. MiRNAs are produced in the nucleus of the cell, and due to their function they remain in the cell (6). Results from other studies have observed the difference in number of bovine miRNA counts, where there are greater number of bovine miRNA sequences when different tissues are compared (15). It is possible the low number of miRNA sequences in serum may be due to communication with other cells, or because they are excess from cellular processes.

It was established that BLV miRNAs were differentially expressed in serum and WBC, between seropositive and seronegative animals. Of the differentially expressed BLV miRNAs, seropositive animals had a higher expression of BLV miRNAs, when compared to seronegative animals. Most differentially expressed miRNAs had a consistent differential expression with an average of 7 log2 fold, with the exception of blv-miR-B2-3p and blv-miR-B2-5p, which had a differential expression averaging 27 log2 fold. These highly differentially expressed miRNAs target host genes unrelated to response to host stress or immune systems process. Blv-miR-B1-5p and blv-miR-B4-5p targeted host genes associated with response to stress and immune systems processes. It is unclear if BLV miRNAs have any role in the development of leukemia in cattle. However, blv-miR-B1-5p, produced by the BLV targets several genes related with development of leukemia in humans. MiRNAs involved in the development of leukemia in humans could also be responsible for the development of the condition in cattle.

## Data Availability Statement

The datasets generated for this study can be found in the NCBI SRA under BioProject accession number PRJNA378560.

## Ethics Statement

This animal study was reviewed and approved by Institutional Animal Care and Use Committee of the National Animal Disease Center, ARS, USDA.

## Author Contributions

EC and JL conceived and designed the experiment. HM analyzed the data. EC and HM interpreted the results. EC wrote the manuscript. HM and JL reviewed and edited the manuscript. All authors contributed to the article and approved the submitted version.

## Conflict of Interest

The authors declare that the research was conducted in the absence of any commercial or financial relationships that could be construed as a potential conflict of interest.
